# *Pseudomonas aeruginosa* intensive care unit outbreak: winnowing of transmissions with molecular and genomic typing

**DOI:** 10.1016/j.jhin.2017.12.005

**Published:** 2018-03

**Authors:** B.J. Parcell, K. Oravcova, M. Pinheiro, M.T.G. Holden, G. Phillips, J.F. Turton, S.H. Gillespie

**Affiliations:** a Ninewells Hospital & Medical School, Dundee, UK; b School of Medicine, University of St Andrews, St Andrews, UK; c Antimicrobial Resistance and Healthcare Associated Infections Reference Unit, Public Health England, Colindale, UK

**Keywords:** Sequencing, *Pseudomonas* spp., Resistance, Outbreak, Water

## Abstract

**Background:**

*Pseudomonas aeruginosa* healthcare outbreaks can be time consuming and difficult to investigate. Guidance does not specify which typing technique is most practical for decision-making.

**Aim:**

To explore the usefulness of whole-genome sequencing (WGS) in the investigation of a *P. aeruginosa* outbreak, describing how it compares with pulsed-field gel electrophoresis (PFGE) and variable number tandem repeat (VNTR) analysis.

**Methods:**

Six patient isolates and six environmental samples from an intensive care unit (ICU) positive for *P. aeruginosa* over two years underwent VNTR, PFGE and WGS.

**Findings:**

VNTR and PFGE were required to fully determine the potential source of infection and rule out others. WGS results unambiguously distinguished linked isolates, giving greater assurance of the transmission route between wash-hand basin water and two patients, supporting the control measures employed.

**Conclusion:**

WGS provided detailed information without the need for further typing. When allied to epidemiological information, WGS can be used to understand outbreak situations rapidly and with certainty. Implementation of WGS in real-time would be a major advance in day-to-day practice. It could become a standard of care as it becomes more widespread due to its reproducibility and lower costs.

## Introduction

*Pseudomonas aeruginosa* is a Gram-negative bacterium that is ubiquitous in moist hospital environments [1], [2]. It is an opportunistic pathogen in immunocompromised patients that causes a wide range of infections [2], [3], [4], [5], [6]. Hospital water can be a source of outbreaks in neonatal units and both adult and paediatric intensive care units (ICUs), colonizing and forming biofilms in water, taps, sinks, toilets, showers and drains [2], [7], [8], [9], [10], [11], [12], [13]. Routes of transmission include environment to patient – either directly from contaminated water or splashes from water outlets, or indirectly from contaminated hands or equipment. Transmission from colonized patients to the environment and between patients can occur during clinical procedures that create aerosols. Infection can be acquired and arise from the patient's own gut microbiota after pseudomonads have been selected out by antibiotics [14]. Multi-drug resistance in *P. aeruginosa* is common, and the mortality rate in invasive infections is up to 29%; therefore, controlling the spread of this organism is important [15], [16].

Differentiating strains is essential to identify routes of transmission of organisms, identify reservoirs and plot potential chains of transmission. Variable number tandem repeat (VNTR) typing, a polymerase-chain-reaction-based method, represents an improvement in speed and reproducibility over pulsed-field gel electrophoresis (PFGE) whilst providing a similar level of discrimination [1], [16]. Turton *et al.* suggested that isolates similar by VNTR with no strong epidemiological links between them should be confirmed by PFGE [1]. Newer methods of whole-genome sequencing (WGS) offer the potential for greater resolution and reproducibility, and may be faster at identifying strains in an outbreak and deducing the lines of transmission. WGS has been used in the investigation of a variety of bacterial outbreaks and, in some instances, has been used for the investigation of pseudomonas outbreaks. To the authors' knowledge, this is the first study to report data comparing the utility of rapid WGS with the current typing methods (VNTR and PFGE) [17], [18], [19], [20], [21], [22], [23], [24], [25], [26], [27].

## Materials and methods

Four patients were identified as colonized or infected with a strain of *P. aeruginosa* with an unusual resistance profile in an ICU at Ninewells Hospital, Dundee between January 2013 and May 2013. A case finding exercise was undertaken using the definition: ‘a sample positive with *P. aeruginosa* resistant to imipenem isolated from a patient admitted to ICU since 2012’. The case finding exercise yielded a further five patients; however, only two patients had isolates that had been stored by the hospital laboratory. As such, six patient isolates were available for further testing. Water (pre- and post-flush samples) was sampled from 14 water outlets in the ICU for *P. aeruginosa* on 16th May 2013 (Table I). Monitoring swabs were also taken from 11 water outlet drains on the same day [domestic service room wash-hand basin (WHB), Bed 7 WHB, Bed 8 WHB, kitchen sink, kitchen drinking water tap, domestic service room sink, ventilator room sink, ICU entrance WHB, ward area WHB 1, ward area WHB 2, Bed 4 WHB]. Caldicott guardian approval was gained in order to protect patient confidentiality and enable appropriate information sharing.

**Table I:** Results of microbiological detection for *Pseudomonas aeruginosa* in water samples (pre- and post-flush) from water outlets in the intensive care unit (ICU)

Source	Pseudomonas count (cfu/mL) pre-flush samples	Pseudomonas count (cfu/mL) post-flush samples
Ice machine	>100	>100
Domestic service room WHB	37	1
Bed 7 WHB	>100	28
Bed 8 WHB	41	0
Kitchen sink	0	0
Kitchen drinking water tap	0	0
Kitchen hydroboil	0	0
Domestic service room sink	0	0
Ventilator room sink	0	0
ICU entrance WHB	0	0
Chilled drinking water dispenser	0	0
Ward area WHB 1	0	0
Ward area WHB 2	0	0
Bed 4 WHB	0	0

WHB, wash-hand basin; cfu, colony-forming units.

### Samples and susceptibility testing

All clinical specimens had been collected during routine care and processed at the Department of Medical Microbiology Laboratory, Ninewells Hospital. Environmental samples from each water outlet drain were incubated aerobically on MacConkey agar at 37°C and *Pseudomonas* CN Selective Agar (Oxoid Ltd, Basingstoke, UK) at 35°C, and examined after 24 and 48 h. VITEK 2 (bioMérieux, Marcy l’Etoile, France) was used for organism identification and antibiotic susceptibility testing using minimum inhibitory concentrations according to the European Committee on Antimicrobial Susceptibility Testing. An external contractor sampled all water outlets for *P. aeruginosa* using pre- and post-flush samples. Water samples were processed by a laboratory approved by the UK Accreditation Service within 4 h of collection.

### VNTR and PFGE

Environmental and patient isolates were sent to the Antimicrobial Resistance and Healthcare Associated Infections Reference Unit, Public Health England, Colindale for typing (VNTR typing at nine loci and PFGE), as described previously [1].

### WGS and phylogenetic analysis

Isolates were stored on beads at −80°C until processed. The cultures were recultured by the Infection Group, School of Medicine, University of St Andrews. DNA was extracted using QIAamp DNA Mini Kit (Qiagen, Hilden, Germany). The quality of the DNA was measured as A280 nm/A260 nm ratio on NanoVue (GE Healthcare, Little Chalfont, UK), and the concentration of double-stranded DNA was assessed using dsDNA BR Kit on a Qubit 2.0 fluorometer (Thermo Fisher Scientific, Waltham, MA, USA). One-nanogram samples of DNA were used to construct the libraries with Nextera XT kit (Illumina Inc, San Diego, CA, USA). The normalized libraries were sequenced using a 2 × 250 pair-end read of a 500-cycle v2 kit on a MiSeq platform (Illumina Inc, San Diego, CA, USA) using a resequencing workflow. The Illumina sequences generated were deposited in the European Nucleotide Archive under the study accession number ERP023446. Using SMALT (Wellcome Trust Sanger Institute; www.sanger.ac.uk/resources/software/smalt/), reads were initially mapped to the chromosome of *P. aeruginosa* PAO1 (accession number AE004091), and single nucleotide polymorphisms (SNPs) were identified as described previously [28]. In addition, the chromosomes of a representative selection of *P. aeruginosa* strains – B136-33 (accession number CP004061), DK2 (CP003149), LES431 (CP006937), LESB58 (FM209186), M18 (CP002496), MTB-1 (CP006853), NCGM2.S1 (AP012280), PA1 (CP004054), PA38182 (HG530068), RP73 (CP006245), SCV20265 (CP006931), UCBPP-PA14 (CP000438), VRFPA04 (CP008739) and YL84 (CP007147) – were used to provide a wider context for the hospital isolates. For each of these additional *P. aeruginosa* strains, artificial 250bp pair-end reads fastq files were generated using a python script. The generated fastq files were mapped along with the outbreak isolates to the chromosome of *P. aeruginosa* PAO1 and SNPs. Recombination was detected in the genomes using Gubbins (http://sanger-pathogens.github.io/gubbins/) [29].

The core genome regions of the PAO1 and UCBPP-PA14 chromosome were defined by human curation using pairwise Blast comparisons with each other and other *P. aeruginosa* strains [30]. The Artemis Comparison Tool was used to visualize the comparisons [31]. SNPs falling inside mobile genetic elements were excluded from the core genome, as well as those falling in regions predicted by Gubbins to have occurred by recombination. Phylogenetic trees were constructed separately using RAxML v7.0.4 for all sites in the core genomes containing SNPs, using a general time reversible model with a gamma correction for among-site rate variation [32], [33]. For a higher resolution phylogeny, ICU isolates that clustered on a branch with UCBPP-PA14 (CP000438) were mapped to this sequence as described above.

## Results

### Epidemiology

The ICU ward had been free from outbreaks in 2013. It was an eight-bed unit of a teaching hospital with approximately 950 acute beds. The infection prevention and control team (IPCT) were initially concerned that four patients were colonized/infected with a strain of *P. aeruginosa* with the same resistance profile between January 2013 and May 2013. All patient isolates were sensitive to gentamicin, ciprofloxacin, piperacillin-tazobactam and ceftazidime, and resistant to imipenem; most patients had received carbapenem treatment at some point during their admission. Patients had a mixture of diagnoses on admission.

Fluids such as bed bath water and endotracheal aspirate (ETA) were disposed of in the wash-hand basins. The IPCT visited the ward and gave advice in line with national guidance for the appropriate disposal of these potentially contaminated fluids. Procedures for the decontamination of two small pieces of equipment – the ventilator flow sensor and temperature probes – were also reviewed. These items were decontaminated by immersion in a sink filled with hot soapy water. This method was discontinued and sporicidal wipes were used after ascertaining their suitability with the manufacturer. The decontamination sink in the back room was found to have crusting on taps; these taps were replaced. This was the sink in which probes were decontaminated. WHBs were supplied by Pillar mixer taps with integral thermostats (Figure A, see online supplementary material). The flow from the tap ran close to the sink drains, causing splashing. Taps with flow straighteners were at risk of contamination by biofilm. To become more compliant with National Health Service building regulations, these were removed and sink basins were replaced to remove overflow drains. The ice machine was supplied by cold water via a flexible hose. This was identified as an area where bacteria could proliferate; the hose was therefore replaced by a Water Regulations Advisory Scheme (WRAS)-approved hose. Localized cleaning of all affected outlets was performed. An increased flushing regimen was introduced to remove any biofilm that was present within the affected outlets. The recommended flushing regimen was twice per day for 2 min at a time. Remediation works were successful as there was no growth of *Pseudomonas* spp. on repeat testing of outlets and water following these changes.

### Antibiotic susceptibility testing

All isolates were confirmed to be *P. aeruginosa*, and five isolates had an indistinguishable antibiotic susecptibility pattern (Patient A, abdominal drain fluid; Patient B, ETA; Patient D, ETA; Patient E, ETA; Bed 8, WHB).

### Environmental investigation

Water (pre- and post-flush samples) was sampled from 14 water outlets in the ICU for *P. aeruginosa* (Table I). Four areas were found to be positive: ice machine (pre- and post-flush), domestic service room WHB (pre- and post-flush), Bed 7 WHB (pre- and post-flush), and Bed 8 WHB (pre-flush). The initial results suggested that the pseudomonas contamination was most likely local to the outlets as the post-flush samples yielded much lower growth results and negative results compared with the pre-flush samples. Monitoring swabs were also taken from 11 water outlet drains, and three were positive (Bed 7 WHB water outlet drain, Bed 4 WHB water outlet drain and ICU entrance WHB water outlet drain).

### VNTR and PFGE analysis

VNTR analysis of the isolates from the ICU identified that six isolates belonged to a cluster of related profiles, which included Patients B and D and the four environmental isolates from Bed 8 WHB water, Bed 4 WHB water outlet drain, Bed 7 WHB water outlet drain and kitchen ice machine water (Table II). All of these isolates had VNTR profiles that were similar to the PA14 strain, one of the most abundant clonal complexes in the *P. aeruginosa* population, which can be readily isolated from aquatic sources causing infections in humans [34]. The close relationship of these isolates in the PA14 cluster suggested that these isolates may be part of an outbreak. In contrast, the isolates from Patients A, C, E and F had distinct VNTR profiles, both from one another and the PA14 cluster, and also from the remaining environmental samples, suggesting that these were unlinked and therefore could be ruled out of the outbreak.

**Table II:** Variable number tandem repeat (VNTR) profiles of *Pseudomonas aeruginosa* isolates from the intensive care unit (ICU)

Source	Date of sampling	VNTR
Bed 4 WHB water outlet drain	16/05/2013	12,2,1,5,5,2,4,5,11
Bed 7 WHB water outlet drain	16/05/2013	12,2,1,5,5,2,4,5,11
Bed 8 WHB water	16/05/2013	12,2,1,5,5,2,4,5,12
Domestic service room WHB water	16/05/2013	12,3,6,3,1,4,14,5,10
ICU entrance WHB water outlet drain	16/05/2013	12,3,-,3,1,4,14,5,10
Ice machine water	16/05/2013	12,2,1,5,5,2,4,5,14
Patient A abdominal drain fluid	11/03/2012	12,6,7,5,3,4,8,1,11
Patient B ETA	21/09/2012	12,2,1,5,5,2,4,5,12
Patient C ETA	04/01/2013	11,2,6,-,3,6,6,6,12
Patient D ETA	15/04/2013	12,2,1,5,5,2,4,5,12
Patient E ETA	11/05/2013	12,4,-,-,3,1,6,4,13
Patient F ETA	05/05/2013	12,2,-,3,2,2,-,5,6

WHB, wash-hand basin; ETA, endotracheal aspirate.

PFGE was used to distinguish the PA14 cluster isolates. Analysis of the banding pattern divided the isolates into three distinct subtypes designated NINE04PA-1 (Bed 8 WHB water, Patient D ETA, Patient B ETA), NINE04PA-1' (Bed 4 and 7 WHB water outlet drain) and NINE04PA-1” (kitchen ice machine water). There were clear and definite band differences between the ice machine isolate and the patient isolates.

### Genome sequencing and phylogenetic reconstruction

**Figure 1:**
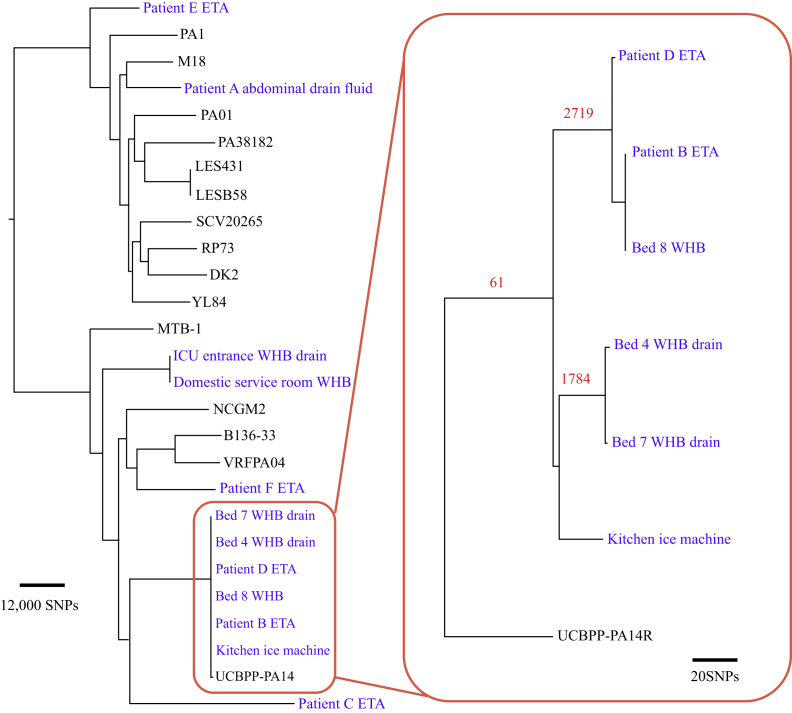
Phylogenomic analysis of *Pseudomonas aeruginosa* isolates. Maximum likelihood phylogenetic tree built with core single nucleotide polymorphisms (SNPs) identified by mapping to the PAO1 reference genome is presented on the left of the figure. The box on the right contains a maximum likelihood phylogeny of the intensive care unit isolates belonging to the PA14 clone, where reads were mapped to the PA14 reference genome of UCBPP-PA14R. The tree was built with core SNPs, excluding SNPs identified in regions that had arisen by recombination (the number of SNPs associated with recombination is given in red text above the branches on which they were identified). Scale bars illustrating the relative SNPs' distances of the phylogenetic trees are displayed. ETA, endotracheal aspirate; ICU, intensive care unit; WHB, wash-hand basin.

WGS and phylogenetic analysis were performed in order to resolve the fine-scale relationship between outbreak isolates and explore epidemiological links between the isolates (Figure 1). In order to provide a wider genetic context and a snapshot of diversity within the species, 15 additional *P. aeruginosa* genome sequences from EMBL nucleotide database were included in the analysis. For this overview of the *P. aeruginosa* population, the WGS reads of the isolates were mapped to the chromosome of the reference strain PA01 [25]. In total, 182,476 SNP sites were identified amongst all analysed isolates and revealed a diverse population structure, throughout which the ICU isolates were distributed. The cluster of isolates identified by VNTR as belonging to the PA14 clone formed a distinct clade in the phylogenetic tree that included the reference isolate UCBPP-PA14, which belonged to the PA14 clone. The next closest isolates to the PA14 cluster were those belonging to Patient C ETA and Patient F ETA, and differed from the cluster by approximately 49,000 SNPs and approximately 50,000 SNPs, respectively.

**Figure 2:**
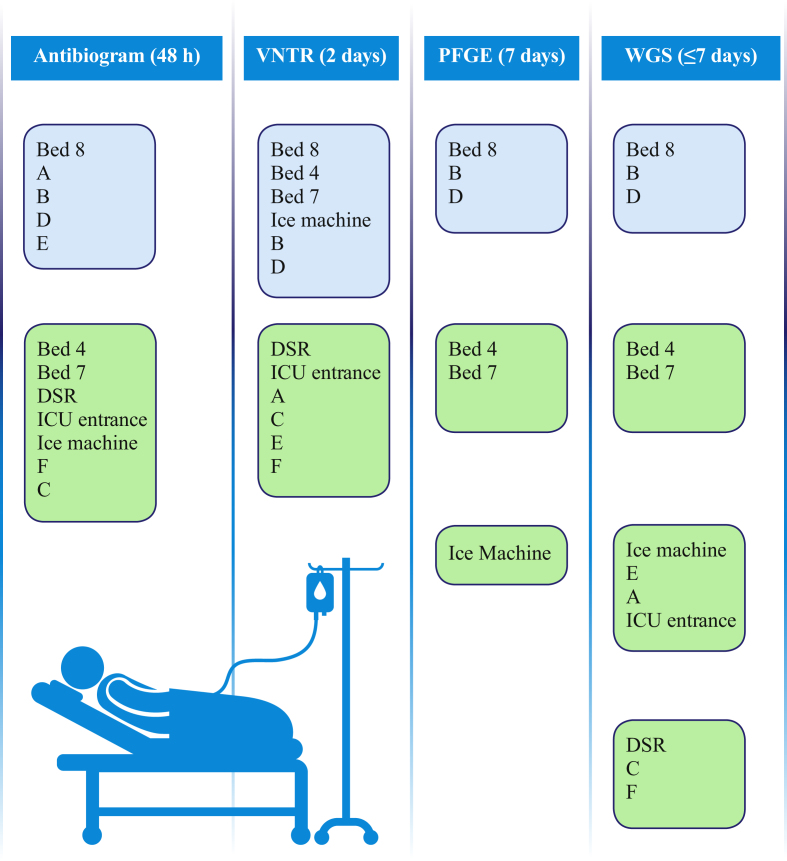
Overview of conventional typing and genomic analysis. Environmental samples: Bed 4, Bed 4 wash-hand basin (WHB) water outlet drain; Bed 7, Bed 7 WHB water outlet drain; Bed 8, Bed 8 WHB water; DSR, domestic service room WHB water; ICU entrance, ICU entrance WHB water outlet drain; ice machine, water. Patient samples: A, Patient A abdominal drain fluid; B, Patient B endotracheal aspirate (ETA); C, Patient C ETA; D, Patient D ETA; E, Patient E ETA; F, Patient F ETA. Light blue, strains deemed ‘in’ by typing; light green, strains deemed ‘out’ by typing. VNTR, variable number tandem repeat; PFGE, pulsed-field gel electrophoresis; WGS, whole-genome sequencing; ICU, intensive care unit.

In order to provide greater resolution for the relationship of the PA14 cluster isolates, the WGS reads were remapped to the reference chromosome of UCBPP-PA14 [26]. This isolate was genetically closer to the outbreak isolates than PAO1, and therefore remapping to this isolate's chromosome would provide increased genomic coverage and consequently greater resolution. Initial phylogenetic analysis of the SNP data mapped to UCBPP-PA14 differentiated the isolates into two separate clusters and a further outlier that were each distinguished by over 1000 SNPs: one cluster containing Patient D ETA, Patient B ETA and Bed 8 WHB water, which was distinguished from the second cluster containing Bed 4 WHB water outlet drain isolate and Bed 7 WHB water outlet drain isolate by 4515 SNPs, which in turn was distinguished from the kitchen ice machine water isolate by 1852 SNPs. Analysis of the distribution of SNPs in the chromosome identified regions of high SNP density, indicative of this variation arising by homologous recombination. Utilizing Gubbins to detect potential regions of recombination identified five regions that distinguished the PA14 clone ICU population. Excluding the SNPs in these regions from the phylogenetic reconstruction reduced the apparent genetic diversity of the PA14 group, but still maintained the distinction of the two clusters and the outlier. In the Patient D ETA, Patient B ETA and Bed 8 WHB water cluster, the Patient B ETA and Bed 8 WHB water isolates were indistinguishable, and differed from the Patient D ETA isolate by four SNPs. The minimal genetic distance between these isolates strongly supports transmission between the Bed 8 WHB water and Patients D and B, and is within the range of SNP distances observed in a study that investigated *P. aeruginosa* transmission in a hospital setting [26]. These patients were not in the ICU department at the same time. An overview of conventional typing and genomic analysis and the timeline for the delivery of these results is illustrated in Figure 2.

## Discussion

The IPCT had urgent questions to answer: is there an outbreak, is there a common source, who is involved, how did the outbreak arise? This study evaluated the relative utility of typing methods to answer these questions. As one moves from routine methods of antibiogram through VNTR and PFGE to WGS, the understanding of the nature of this outbreak becomes apparent. There is a progressive winnowing of possibly involved patients and infection sources. The antibiogram showed a linkage between the Bed 8 WHB water and Patients B and D, but also included Patients A and E. VNTR correctly identified the two patients who were part of the outbreak, but also identified several false-positive environmental links. This left the IPCT considering various routes of transmission; for instance, patient care using ice machine water. PFGE was required for complete clarification. The clinical benefit of using WGS in this situation is that it rapidly provides absolute clarity in distinguishing the isolates in one step, negating the need for the IPCT to spend unnecessary time contemplating other scenarios of how the transmission came about without certainty. In this situation, this information was combined with epidemiology. These patients were not in the ICU at the same time, suggesting that the water supply had acted as a reservoir and source of ongoing infection.

Recognized interventions to prevent transmission from water to patients were effective in preventing further transmission. These included removal of taps with flow straighteners, replacement of sink basins with overflow drains, introduction of increased flushing regimen, and monitoring of water temperature to become fully compliant with national guidance. The IPCT also reviewed procedures for the decontamination of the ventilator flow sensor and temperature probes, in addition to making recommendations for the disposal of potentially contaminated fluids to prevent transmission of organisms from patients to sink drains and distal ends of taps. ICU staff supported changes to their decontamination practices, and training on the new cleaning protocol for sinks was also given. Following this, areas were resampled and it was confirmed that remediation works were successful.

This study has shown that WGS is a potent tool to direct effective intervention in outbreak situations in comparison to, and providing additional information to, molecular typing methods. WGS can provide results within seven days; however, it should be noted that current start-up costs for this technology remain high. For WGS to be introduced into routine clinical microbiology laboratories, investment in infrastructure including bioinformatics and expertise for the interpretation, management and storage of data is required. Standard operating procedures, validation of methods and quality control measures are in place for VNTR and PFGE testing, and will be required for WGS to take place in clinical laboratories. The results are limited by the fact that some isolates were not stored and not all were recultured successfully. Only one colony was processed from each sample, and this may have limited assessment of the diversity of *Pseudomonas* spp. in patient and environment samples.

WGS would be of particular use when there are no obvious epidemiological links between the patients, enabling IPCTs to have timely results using one method. WGS alone provided the necessary resolution to identify the transmission pathway, demonstrating unequivocally the spread between single water supply to patients, and eliminating other potential transmission events and sources. Thought should be given as to how to make these powerful data available routinely in a timely manner, and in a format that is easily interpretable and clinically relevant. Establishing tools such as sequencing machines locally can reduce the turnaround time. It is essential that clinicians develop a new approach to investigate hospital outbreaks, and escalate to WGS at an early stage to allow accurate and rapid description of the causes. It is only by using WGS in real-time that it will be used as a powerful tool to improve patient outcomes.

## Figures and Tables

**Figure A:**
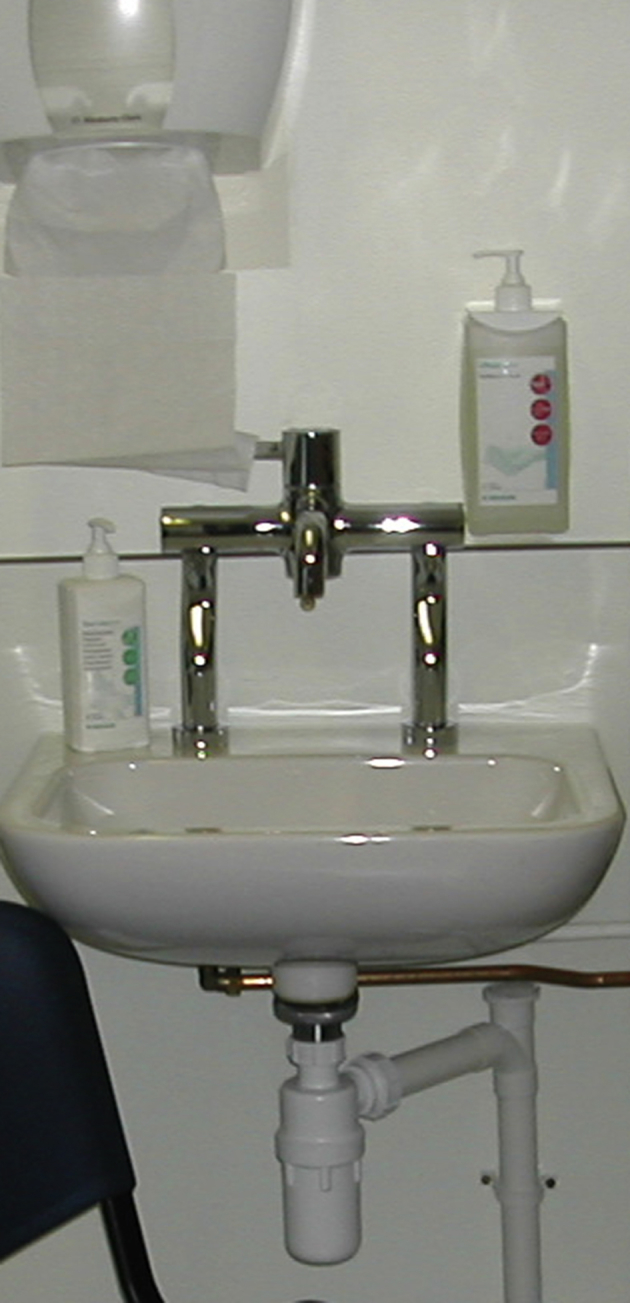
Example of the type of sink that was used on the intensive care unit at the time.
